# Osteoarthritis of the hip: is radiography still needed?

**DOI:** 10.1007/s00256-022-04270-8

**Published:** 2022-12-20

**Authors:** Charbel Mourad, Bruno Vande Berg

**Affiliations:** 1 Department of Diagnostic and Interventional Radiology, Hôpital Libanais Geitaoui CHU, Beyrouth, 1100 Achrafieh Lebanon; 2 Department of Radiology, Cliniques CHC Montlégia, Boulevard Patience Et Beaujonc 2, 4000 Liège, Belgium

**Keywords:** Hip, Conventional radiography, Osteoarthritis, MRI

## Abstract

Diagnosis of hip osteoarthritis (OA) is based on clinical arguments, and medical imaging is obtained to confirm the diagnosis and rule out other possible sources of pain. Conventional radiographs are recommended as the first line imaging modality to investigate chronic hip pain. They should be obtained in a rigorous technique that includes an antero-posterior (AP) radiograph of the pelvis. The choice of the appropriate lateral view depends on the clinical indication, Lequesne’s false profile being valuable in the assessment of OA. Magnetic resonance imaging (MRI) is more sensitive to detect joint effusion/synovitis, cartilage, labral, and bone marrow lesions. However, structural joint changes are frequent in asymptomatic population and neither radiographs nor MRI have shown a good correlation with pain and functional impairment. MRI seems to be more suitable than radiographs as a biomarker for clinical trials addressing early OA. The absence of a validated MR biomarker of early OA, together with issues related to machine availability and MRI protocol repeatability, prevent the widespread use of MRI in clinical trials.

## Introduction

Hip osteoarthritis (OA) is a highly prevalent and disabling disorder that affects elderly but also young patients with a high socio-economic burden [1–7]. In patients with clinically suspected hip OA, medical imaging contributes to confirm the diagnosis and rule out alternative diagnoses by demonstrating cartilage lesions and associated structural changes [8]. For decades, conventional radiography (CR) has been used to support the clinical diagnosis of hip OA. Over the years, magnetic resonance imaging (MRI) emerged as a powerful imaging modality to detect cartilage lesions and structural changes of the hip joint. The current special issue of Skeletal Radiology granted us the opportunity to address a fundamental question: is radiography still needed to diagnose hip OA? Which imaging modality should be used to diagnose stage and quantify hip OA in clinical practice, in clinical trials and in research? After a brief review on classifications and diagnostic criteria, the current narrative article will summarize strengths and weaknesses of CR and MRI to diagnose hip OA and will propose perspectives on the use of medical imaging. This review article also highlights the importance of rigorous acquisition and reading of hip radiographic and MR images. Imaging of femoro-acetabular impingement (FAI) and advanced quantitative MR techniques for the cartilage are out of the scope of this article and will be addressed separately in this Skeletal Radiology issue.

## Classification systems of hip osteoarthritis

**Fig. 1:**
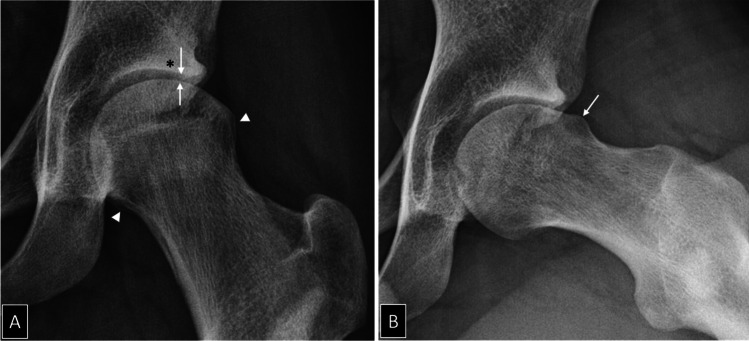
A 21-year-old man with moderate left hip pain and osteoarthritis secondary to femoro-acetabular impingement. **A** AP radiograph demonstrates lateral joint space narrowing (arrows), subchondral sclerosis of the acetabular roof (asterisk) and femoral head osteophytes (arrowheads). **B** The 45° Dunn lateral view demonstrates Cam deformity at the head-neck junction (arrow)

**Fig. 2:**
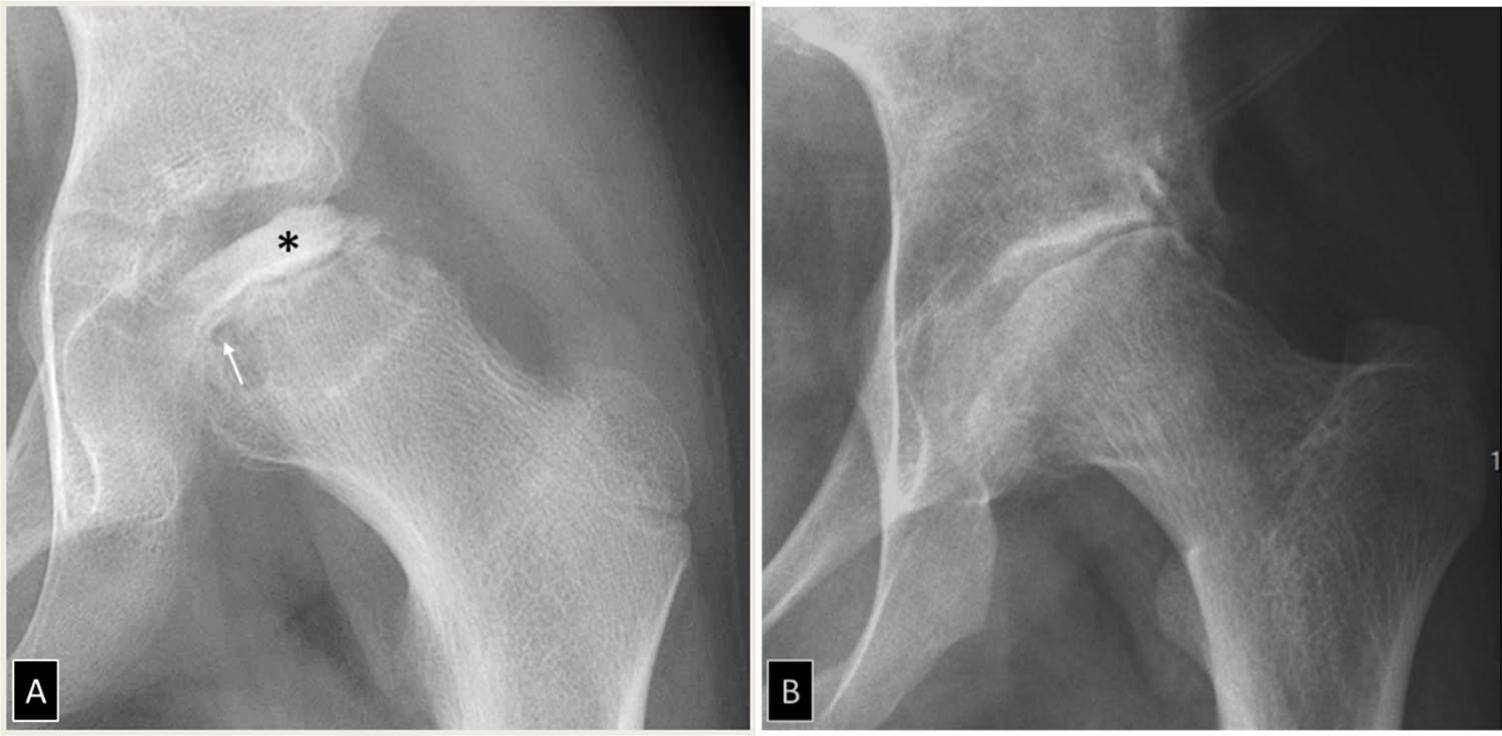
Secondary hip osteoarthritis. **A** AP radiograph of the left hip in an 8-year-old boy with Legg-Calvé-Perthes disease showing sclerosis of the femoral head epiphysis (asterisk) and cystic changes of the metaphysis (arrow). **B** Follow-up AP radiograph of the same patient at the age of 23 years showing secondary osteoarthritis with abnormal femoral head contours, articular incongruity, and joint space narrowing

**Fig. 3:**
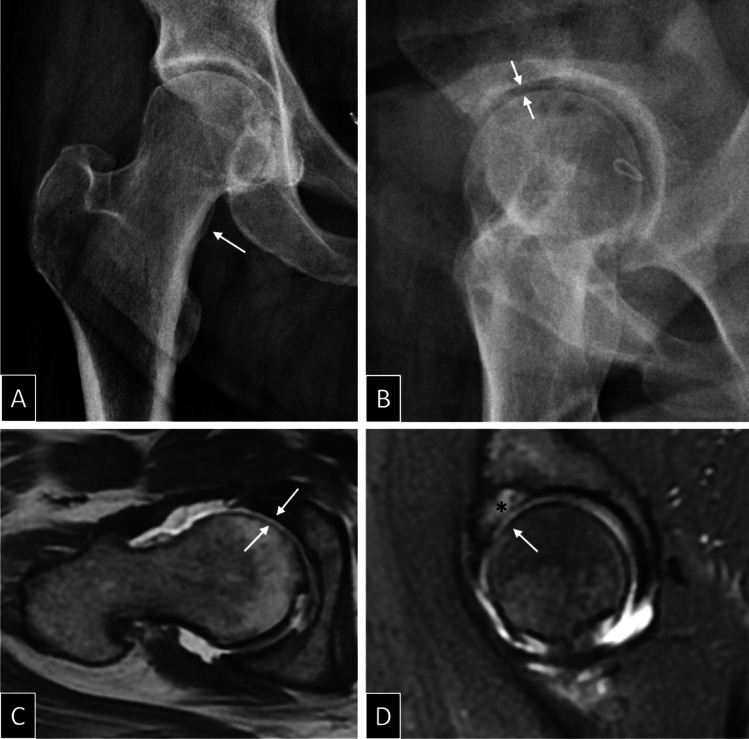
A 38-year-old woman with right hip pain and osteoarthritis. **A** Anteroposterior radiograph shows cortical buttressing (arrow) with a preserved joint space width (Kellgren 1). **B** Lequesne false profile shows narrowing of the antero-superior joint space (arrows). **C** Transverse T1 and **D** sagittal intermediate-weighted fat suppressed MR arthrography images after intra-articular contrast injection show full-thickness cartilage substance loss in the anterior (arrows in **C**) and superior (arrows in **D**) aspect of the joint space, with subchondral cyst-like changes (asterisks in **D**)

**Fig. 4:**
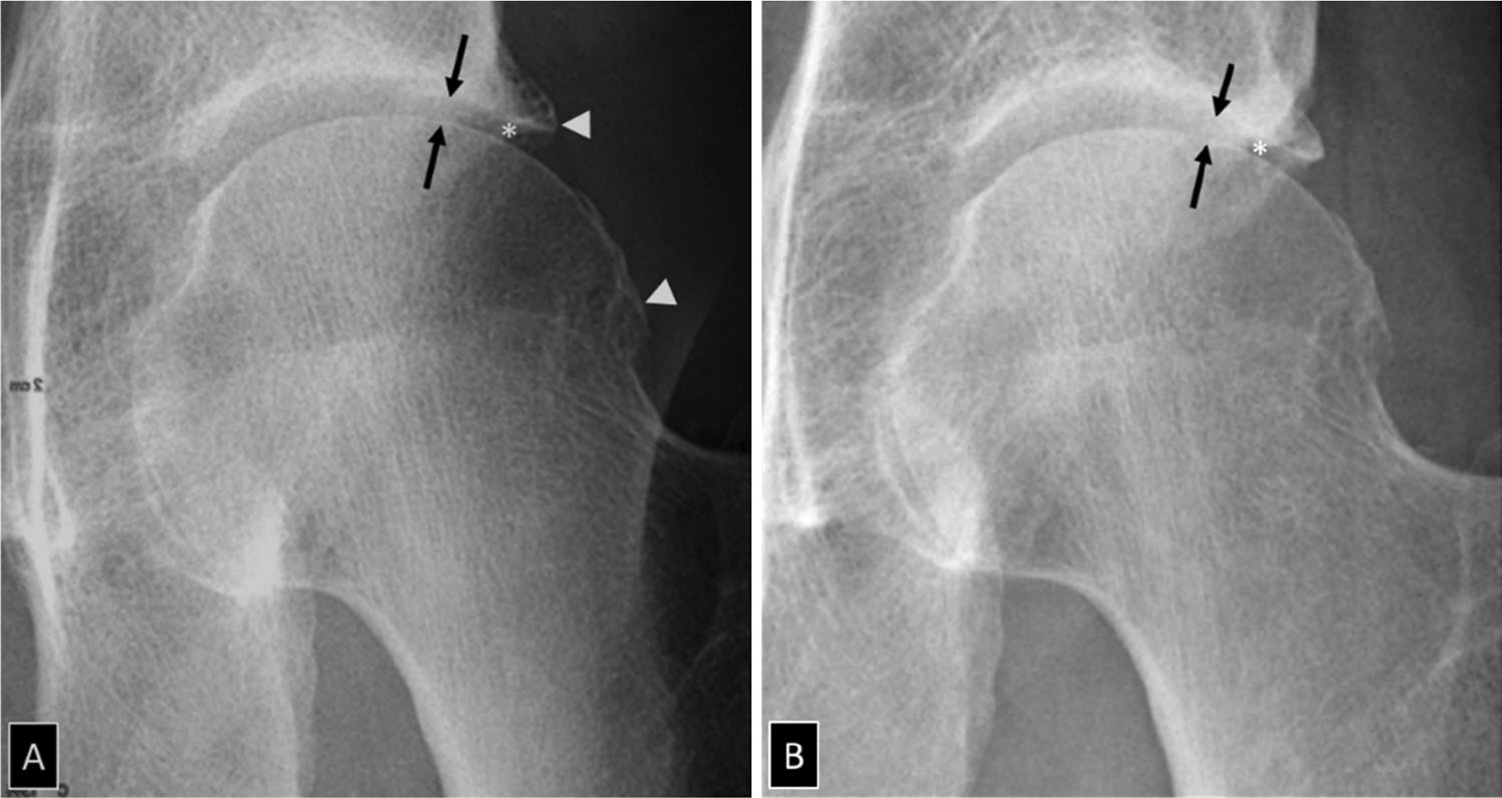
Non-evolutive osteoarthritis of the left hip in a 62-year-old man with limited range of motion but no hip pain. **A** AP radiograph of the left hip demonstrates lateral joint space narrowing (arrows) and marginal osteophytes (arrowheads). **B** Follow-up radiograph after 10 years demonstrates no significant change in joint space narrowing. Outcome prediction on radiographs is unreliable. Note that the area underneath the osteophyte (asterisk) does not correspond to the articular joint space

**Fig. 5:**
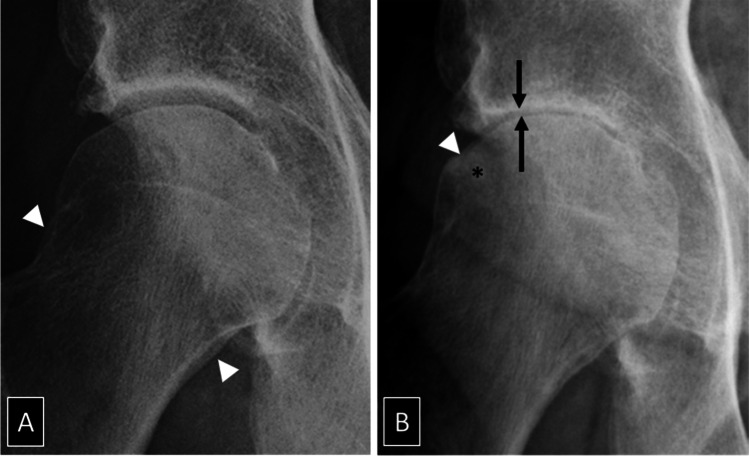
65-year-old man with rapidly destructive osteoarthritis. **A** AP radiograph of the right hip obtained at onset of symptoms shows early osteophytes (arrowheads) but no joint space narrowing. **B** AP radiograph obtained 3 months later shows complete joint space narrowing (arrows) with deformity of the femoral head (arrowhead) and subchondral sclerosis (asterisk).

Hip OA can be classified according to its etiology, time of onset, severity, and clinical course. In primary hip OA, cartilage degradation can either be idiopathic or develop in association with dynamic conflict between the articular surfaces, the FAI syndrome [9–11] (Fig. 1). In secondary hip OA, joint degradation results from preexisting conditions including developmental hip dysplasia, growth-associated disorders, fracture, femoral head osteonecrosis and inflammatory or metabolic synovial disorders [10, 12–17] (Fig. 2). Early-onset and late-onset disease develop either before or after 50 years of age [2]. Early-stage and late-stage OA differ according to the absence or presence of radiological structural changes with a joint space width (JSW) of more or less than 2 mm on AP pelvic radiographs [18–20] (Fig. 3). The clinical course of hip OA is usually slow and pain fluctuates over the years with no or minor radiological changes over time [21] (Fig. 4). Rapidly destructive hip OA is uncommon and is defined by the development of complete loss of radiological JSW or severe bone attrition on CR within 12 months after symptoms onset [22–24] (Fig. 5). All these classification systems and threshold values are open for discussion, but they rely on good clinical practice standards for which preservative hip surgery should not be performed after 50 years of age or when the radiological JSW is < 2 mm.

## Clinical diagnosis of hip osteoarthritis

### Clinical history

Typically, OA-associated hip pain evolves over time with initial intermittent activity-related or weight-bearing pain followed by constant pain, limited range of motion, and altered gait. Several population- or OA-based cohort studies have shown that, on average, there is little to no progression of complaints during a 10-year follow-up period [21, 25] Hip pain can be localized anteriorly, laterally or posteriorly but it may also be referred in the groin, the buttock, the thigh or the knee. A major clinical challenge is to recognize articular hip pain from peri-articular or non-hip-related pain [26–31].

## Physical examination

Physical examination remains one of the most valuable tools physicians can use to diagnose hip OA. Upright and supine exam tests as well as provocative maneuvers have been developed to increase the likelihood that presenting symptoms originate from the hip joint [32–35]. Unfortunately, the accuracy of these tests varies with a wide range of sensitivity and specificity [33]. For example, the flexion-abduction and external rotation test had a sensitivity ranging from 41 to 97% and a specificity ranging from 18 to 100% [36]. Therefore, the specific application and interpretation of these clinical tests must be integrated in the context of the patient’s history.

## Biological tests

Routine blood tests play little diagnostic role in hip OA, but they contribute to rule out alternative diagnoses. The American College of Rheumatology criteria provide a set of clinical, laboratory, and/or radiographic features to identify patients with OA and to distinguish them from patients with other diseases [37]. Many biological markers may be altered in severe hip OA as in inflammatory or immune-mediated articular disorders, but their use remains limited to research setting and are not used in clinical practice [38, 39].

## Radiological diagnosis of hip osteoarthritis

### Radiographic hip examination

Radiological workup of the hip includes at least an antero-posterior (AP) radiograph of the pelvis and a lateral radiograph of the hip. The pelvic radiograph provides an overview of the entire pelvic girdle and allows a comparative analysis of both hips, which enhances the detection of subtle bone and joint abnormalities [8, 40–43]. The pelvic AP radiograph should also be used to assess FAI-associated features since pelvic positioning can be controlled on the AP pelvic radiograph but not on the AP hip radiograph [44]. There is no clear consensus whether the JSW is better evaluated on weight-bearing (WB) or non-weight-bearing (nWB) AP pelvic radiographs [45–48]. The WB radiograph evaluates the pelvis in its anatomical position with a decrease in acetabular coverage and increase in posterior pelvic tilt in comparison to the nWB pelvic radiograph [49]. The WB radiograph has an overall decrease in image quality with a higher radiation dose, is less reproducible and does not provide additional joint space narrowing (JSN) than nWB radiographs, except in severe acetabular dysplasia and advanced OA [18, 50–52].

**Fig. 6:**
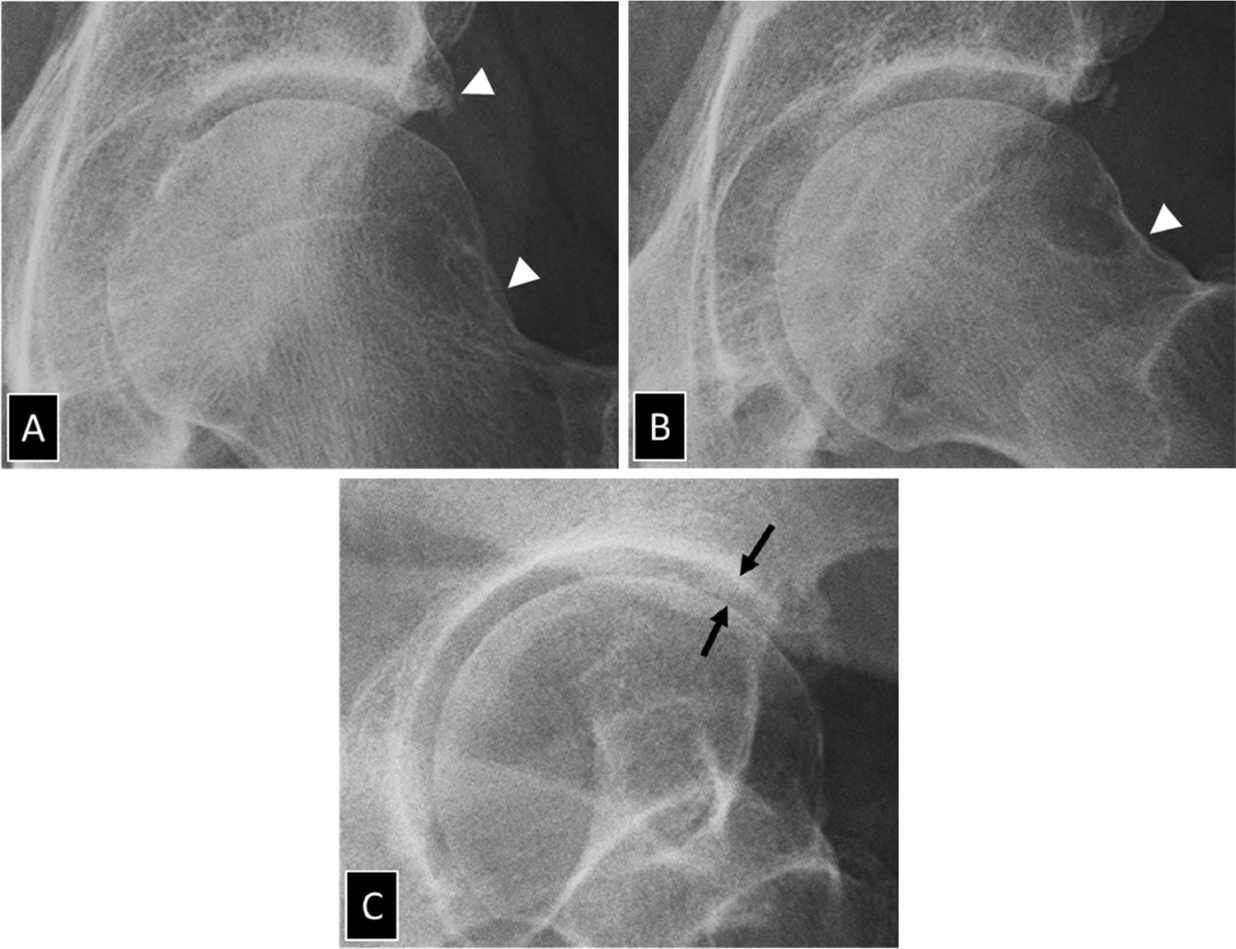
Value of Lequesne’s false profile view in a 65-year-old man with mild hip pain after walking. **A** AP and **B** Lauenstein lateral radiographs demonstrate almost normal joint space and osteophytes (arrowheads). **C** Lequesne’s false profile demonstrates almost 50% joint space narrowing in the anterior aspect of the joint (arrows) that cannot be seen on the other radiographs

Lateral radiographs of the hip can be obtained with different degrees of pelvic rotation, hip abduction and flexion [40–42]. They provide variable lateral views of the proximal femur [42, 53]. In the setting of early-onset hip OA, the 45° Dunn lateral view offers the better view of the femoral-neck junction to measure Cam-associated features [44, 54, 55] (Fig. 1). The off-lateral view or false profile of Lequesne is the unique radiograph that provides an evaluation of the hip joint in a physiological position in a near sagittal plane [56, 57] enabling to assess anterior acetabular coverage and anterior or posterior JSW [58] (Fig. 6). The off-lateral view enables to detect more hips with JSN than the AP pelvic radiograph alone [58, 59].

### Normal hip radiograph

Radiographs of a normal hip joint may demonstrate some cortical irregularities and labral ossifications that should not be confused with OA-related structural bone changes [41, 60, 61]. The radiographic JSW corresponds to the distance between the acetabular roof and the femoral head, reflecting the combined thickness of the acetabular and femoral head cartilages [62]. Normal JSW varies from 2 to 7 mm among individuals with limited variability (< 1 mm) between both hips of the same individual [63]. The distance between the fovea capitis and the teardrop does not reflect articular cartilage thickness [62]. On a normal pelvic radiograph, the lateral JSW is larger than or equivalent to the medial JSW in 85% and 15% of cases, respectively [60]. The lateral JSW should not be thinner than the medial JSW except in arched acetabular roof and in ossified labrum [60]. On the Lequesne false profile view, the anterior JSW should always be larger than the posterior JSW [57, 64].

### Abnormal radiographic joint space

Joint space narrowing (JSN) of the hip is the radiographic hallmark of late-stage hip OA but is absent in early-stage hip OA. Several patterns of predominant JSN have been recognized, most likely reflecting uneven advanced cartilage loss [62, 65, 66]. Predominant lateral or anterior JSN is the most frequent pattern and is associated with the CAM-type FAI. Predominant medial or posterior JSN is associated with the Pincer-type FAI [62, 66]. Predominant isolated supero-medial JSN can occasionally be seen in late-onset OA and in some secondary forms of hip OA. Good reproducibility and repeatability in the assessment of absent, possible, or definite JSN has been consistently demonstrated but the reproducibility can be influenced by methodological features [48, 67–70].

JSW can be reliably measured by using manual or computer-assisted methods, providing a continuous variable for cartilage evaluation [67, 69, 71]. JSW shows important inter-individual variations that limit its value to compare patients [63]. Serial JSW measurement can be used to track cartilage changes over time, and disease progression was defined as loss of JSW of ≥ 0.5–0.6 mm/year [48, 69, 72, 73]. JSW change over time seems to better correlate with hip symptoms than absolute JSW [67, 71, 73].

### Radiographic structural changes

Structural bone changes on radiographs include subchondral sclerotic or cystic bone changes and osteophyte formation at the periphery of or at distance from the cartilage-covered articular surfaces. Osteophyte formation has received most attention in OA and is an important finding in Kellgren-Lawrence scoring system [74–79]. There is currently no accepted or validated definition for early OA [18]. In many clinical trials, hip OA is defined by a Kellgren-Lawrence grade ≥ 2 (definite JSN, definite osteophytes, and possible sclerosis) [74, 75, 80]. The strengths and weaknesses of these grading systems have been extensively addressed [81, 82].

## MRI diagnosis of hip osteoarthritis

### Hip MRI examination

The standard MRI protocol of the hip usually includes fat-sensitive and fat-saturated fluid-sensitive sequences with the highest spatial resolution achievable which is higher at 3.0 than at 1.5 Tesla. Recommended imaging planes vary and include standard or oblique coronal, sagittal and transverse planes, radial imaging and 3D imaging [83–85]. Direct hip traction MR arthrography can also be performed for dedicated cartilage and labrum evaluation but is more invasive than standard MRI due to the need for articular puncture and hip traction.

### MRI of the hip cartilage

In fact, MRI of hip cartilage reached maturity later than that of knee cartilage due to several technical challenges that are specific to the hip. Meta-analyses demonstrated a lower accuracy of conventional MRI for the detection of cartilage defects at the hip than at the knee [86, 87]. Technical challenges for hip cartilage MRI include (a) deep and eccentric location of the hip, (b) absence of dedicated hip coils (c) thinness of the hip cartilage, (d) high congruency of the articular surfaces with no fluid between the two hyaline cartilage surfaces [18, 88], and (e) complex anatomy with partial volume artifacts. MRI can demonstrate focal morphological changes of the cartilage, like signal alteration, substance loss, and delamination, before radiographic JSN occurs [89, 90] (Fig. 3). Cartilage defect may represent early biomarker for OA [89]. Compositional and quantitative MRI techniques of the cartilage add some insights into the sequence and rate of articular cartilage changes at the hip that precede overt hip OA [91].

### MRI structural changes in hip osteoarthritis

Besides the depiction of hyaline and labral cartilage changes, conventional MRI depicts structural bone changes including bone marrow edema (BME)- and sclerosis-like signal changes along with joint effusion and synovitis [89, 92–95]. Osteophytes and subcortical cysts are more conspicuous at MRI than at CR because of its multiplanar capacity. MRI, a powerful diagnostic tool in OA imaging, has dramatically changed our understanding of OA that evolved from a cartilage-centered disease to a whole joint organ disease. This new approach of OA offers potentials for early diagnosis and outcome measures for new treatments [90, 94]. However, there is currently no accepted or validated definition of hip OA based on MRI [18]. Several semi-quantitative scoring systems based on location and severity of articular changes have been developed and validated for hip MRI in clinical trials and research including (1) Scoring Hip osteoarthritis with MRI (SHOMRI), (2) Hip OA MRI Scoring System (HOAMS), and (3) Hip Inflammation MRI Scoring System (HIMRISS) [94, 96–100].

## Strengths and weaknesses of clinical, radiographic and MRI hip examinations in the setting of suspected hip osteoarthritis

### Clinical examination

Clinical examination of the hip of patients with suspected early-stage hip OA is feasible, available, and repeatable with a moderate interobserver reproducibility [101]. Its accuracy is acceptable when findings are integrated with past medical history and present clinical history, in the absence of clinical red flags [33, 102]. Exclusion of all red flags is mandatory to accept a presumptive diagnosis of hip OA [45]. A major weakness of clinical examination is its low sensitivity for detecting early-onset and early-stage hip OA. Clinical examination does not provide any staging system or predictive information but can be used to monitor disease progression (Table 1).

**Table 1:** Strengths and weaknesses of clinical, radiographic and MR examinations of the hip in patients with suspected hip OA based on authors’ opinions

	Clinical examination	Radiographs	MRI
Feasibility			
Patient acceptability	+ + +	+ + +	+
Contra-indications	−	−	+
Availability	+ + +	+ + +	+
Reproducibility	+ +	+ +	+
Short-term repeatability	+ + +	+ + +	+ + +
Long-term repeatability	+ +	+ + +	+
Radiation	−	+	−
Cost	−	+	+ + +
Comparative hip joint evaluation	+ + +	+ + +	+
Self-confidence of clinicians in primary interpretation	NA	+ + +	+
Diagnostic performance			
Sensitivity for OA	−	−	+ + +
Specificity for OA	+	+ + +	+ +
Negative predictive value for OA	−	−	+ + +
Positive predictive value for OA	+	+	+ + +
Focal cartilage lesion	−	−	+ +
Diffuse cartilage lesion	+	+ +	+
BME	−	−	+ + +
Labral tear	+	−	+ +
Subchondral cyst	−	+	+ + +
Osteophytes	−	+ +	+ + +
Joint effusion, synovial swelling	−	−	+ + +
Quantitative parameters	+	+	+ + +
Correlation between imaging findings			
Pain	NA	+	+ +
Range of motion	NA	+	+ +
Provocative maneuvers	NA	−	+ +
Impact on therapeutic decision/management			
CAM-associated features	+	+	+ + +
Pincer-associated features	+	+ +	+ + +
Femoral torsion	−	−	+ +
Planning for total hip replacement	−	+ +	−

### Radiographic examination

Radiological examination of the hip of patients with suspected OA is feasible, available, and repeatable with an acceptable moderate interobserver reproducibility [67, 103]. Despite some variations in radiological practices, the radiographic technique is well documented and reproducible among institutions and over time. Long-term follow-up radiographs can be compared with initial films. Contra-indications for pelvic radiographs are negligible. In the setting of hip OA, pelvic radiographs are easily interpreted by radiologists and clinicians. It yields valuable information to differentiate primary from secondary OA and to detect FAI-associated anatomical features.

Pelvic radiography has poor sensitivity in the detection of many soft tissue, bone, and joint changes [104, 105] and therefore has limited value for ruling out alternative disorders. In the setting of hip OA, radiographs are insensitive to compositional and early structural changes; JSW measurement is insufficient to assess articular cartilage. Deep cartilage defects can be observed at MRI despite normal radiographic JSW of that hip [89] (Fig. 3). Given these limitations, CR does not fulfill the mandatory requirements to become a valuable biomarker of early OA in clinical trials [80, 95, 103, 106].

**Fig. 7:**
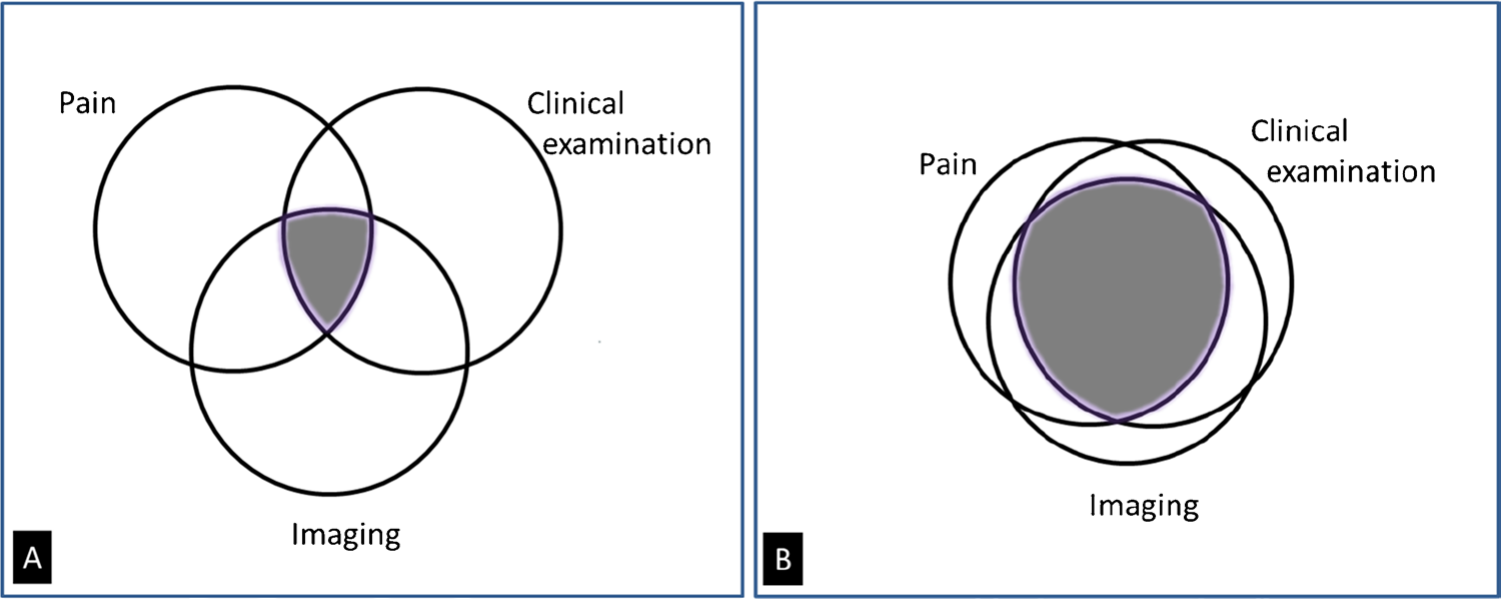
Hypothetical Venn diagram illustrating the lack of coherence between pain, clinical examination, and imaging findings in patients with **A** presumed hip osteoarthritis and **B** femoral neck fracture. The shaded area represents the proportion of patients in whom all three parameters are present. In OA, discordance between symptoms, clinical examination, and imaging findings are frequent. In femoral neck fractures, the three parameters are overlapping

Radiological-clinical discordances have been frequently observed; hips with radiographic OA may remain asymptomatic and, conversely, painful hips due to early OA may not show radiographic signs of OA [107, 108] (Fig. 7). Intensity of symptoms fairly correlates with radiological staging of OA [109, 110]. In addition to these radio-clinical dissociations at an early stage, radiographic hip OA progression poorly correlates with pain progression [111]. Early radiographic changes also lack predictive values for the development of clinical hip OA at 5–10 years follow-up in large patients cohorts [25, 112]. Change in JSW seems to better correlate with symptoms than the absolute JSW at a given moment (Table 1).

### MRI examination

Hip MRI is worthwhile feasible with some limitations in availability. In the setting of early OA, a high-resolution hip MRI examination is needed and is the most accurate imaging modality to rule out alternative diagnoses and to diagnose and stage hip OA. Hip MR arthrography with hip traction is more accurate than standard MRI for the depiction of cartilage changes indicating some limitations in the accuracy of non-arthrographic MRI [113]. The interpretation of hip MRI by non-expert radiologists and clinicians remains to be validated in the setting of OA. Variations in local radiological equipment and practices may limit its use in large multi-center studies. Constant improvement in image quality, the availability of new MR sequences and advanced image post-processing might represent a challenge when evaluation long term hip changes at future MRI examinations.

MRI clinical discordances have been seen. Hips with cartilage lesions at MRI may remain asymptomatic, and symptomatic hips may show no signs of OA at MRI [114]. OA-related MRI lesions can also be observed in asymptomatic volunteers [114, 115]. Some hip changes moderately correlate symptoms, including femoral head BME, synovitis/effusion, and muscle atrophy [93, 116]. The severity of BME could correlate that of pain and number of microfractures [93]. to the best of our knowledge, the prevalence and rates of progression of hip changes in OA have not been established yet at MRI [91]. It is therefore not surprising that some clinical trial organisators are reluctant in introducing MRI at inclusion or as a biomarker due to limitations in its interpretation and/or overlapping findings between symptomatic and asymptomatic hips (Table 1).

## A plea for CR as first-line imaging modality in clinically suspected hip osteoarthritis

Rising healthcare costs is a major concern in both the political and medical professions with diagnostic imaging representing one of the fastest rising cost segments worldwide. There is a need for value-driven diagnostic algorithms and decreasing unnecessary diagnostic testing that may not alter the course of patients’ management can efficiently reduce healthcare costs [117].

In patients aged above 50 years and hip symptoms suggestive of OA, scientific associations recommend to obtain pelvic and hip CR as the first-line imaging modality [8, 27, 45, 102, 118–121]. However, cost-effectiveness studies assessing the value of hip radiographs in patients with suspected hip OA indicated a variable impact on patient’s management. As a matter of fact, a presumptive clinical diagnosis of hip OA could be accepted without requiring medical imaging in patients > 50 years without previous relevant medical history as long as symptoms and clinical examination at onset and clinical course at follow-up remain consistent with this diagnosis [45]. A trend in a more comprehensive approach of patients with age-associated articular pain further supports a declining role of medical imaging, in the absence of red flags at onset and of unexpected clinical evolution. A recent study demonstrated that psychological and behavioral characteristics of patients with hip pain better correlated with hip pain than any arthroscopically demonstrated hip lesion [122]. To the best of our knowledge, we are not aware of any validated recommendation proposing hip MRI as the first imaging modality for suspected hip OA in patients aged >50 years. Cost-effectiveness studies in several MSK disorders including OA assessing the value of MRI on patient management demonstrated a significant increase in cost without any therapeutic effect [117, 123].

In patients aged below 50 years of age and presumed hip OA, numerous scientific associations also recommend to obtain conventional radiographs of the hip as the first-line imaging modality without any delay [45, 55, 120, 124, 125]. Studies assessing cost-effectiveness of radiographs are lacking as hip radiographs are used for patient inclusion. Currently, the medical community is still awaiting the multi-center confirmation that preservative hip surgery in young patients with FAI-related disorders is a disease modifying intervention. In case of reliable positive results, MRI could be obtained as second-line imaging modality if MRI yields independent markers to select patients who would benefit from surgery. Cost-effectiveness analysis of hip MRI in the setting of FAI will then become mandatory.

## Perspectives that would favor the use of MRI as first-line imaging modality

A currently not foreseen increase in MRI equipment availability and decrease in examination time duration and cost would be mandatory to significantly change patient’s imaging workflow. Currently, in the absence of disease modifying drugs, healthcare system efforts focus on tertiary prevention of hip OA to soften the clinical and functional consequences and to postpone total hip replacement. Currently, there are no proven disease modifying drugs approved by the regulatory agencies and, therefore, the impact of early detection of OA is limited. The availability of disease modifying drugs and of MRI biomarkers that would enable to select patients who are likely to benefit from these drugs would definitely support the use of MRI as a screening tool. Cost-effectiveness studies would then be of value once the cost of these disease modifying drugs would be determined. (Tables 2 and 3).

**Table 2:** Factors that support radiographs as first imaging modality in suspected hip OA

• Lack of availability of disease-modifying drugs
• High prevalence of OA
• Limited diagnostic value of clinical history and examination
• Readability by non-radiologists
• Weaknesses of MRI

**Table 3:** Factors that need to be fulfilled before abandoning radiographs as first imaging modality in suspected hip OA

• Availability of disease-modifying drugs
• Validation of long-term results of disease-modifying interventions
• Better availability of MRI (cost, duration, readability)
• Optimized non-contrast enhanced MR images
• Better understanding of mechanisms of pain

Currently, hip MRI is considered to be the most sensitive non-invasive imaging modality that enables to assess local pre-OA changes and is the best imaging modality to assess time- and drug-related changes in radiological studies. However, clinical trial organisators are reluctant in including hip MRI in large clinical trials partly because MRI biomarkers are also found in asymptomatic subjects. The lack of a consensus on what represents early hip OA severely limits the feasibility of clinical trials with difficulties in creating homogeneous patients’ subsets for the purpose of clinical studies. Time has come to assess the value of MRI biomarkers, e.g., cartilage volume and composition, effusion-synovitis, and bone marrow edema-like signal intensity, in population-cohorts’ studies.

## Conclusion

Hip OA is a heterogeneous group of hip disorders with fluctuating symptoms over time and slow progression of radiographic changes. Pelvic/hip CR is the recommended first-line imaging modality to be obtained in the setting of suspected hip OA with some debate on the moment at which it should be performed during the disease course. MRI is the gold standard imaging technique in early OA. The absence of accepted or validated reference standard for early-stage hip OA, the presence of dissociation between symptoms, clinical evaluation and imaging findings along with absence of disease-modifying drugs in hip OA remain challenging in clinical and research practice. CR should be obtained at inclusion in clinical trials including patients with early-stage OA, but possibly has limited outcome significance. MRI has a lot to offer both at patient inclusion and as primary or secondary outcome biomarker.

## Abbreviations

AP: Antero-posterior
BME: Bone marrow edema-like
CR: Conventional radiography
FAI: Femoro-acetabular impingement
JSN: Joint space narrowing
JSW: Joint space width
MRI: Magnetic resonance imaging
nWB: Non-weight-bearing
OA: Osteoarthritis
WB: Weight-bearing
